# Integrative Application of Transcriptomics and Metabolomics Reveals Molecular Insight into Metabolomic Variations in Chinese Mitten Crab *Eriocheir sinensis* Harvested from Lake Datong and Adjacent Pond

**DOI:** 10.3390/biology15020110

**Published:** 2026-01-06

**Authors:** Lehe Lin, Yiming Pang, Wengang Xu, Chun Wang, Huafeng Zou

**Affiliations:** 1 National Demonstration Center for Experimental Fisheries Science Education, College of Fisheries and Life Science, Shanghai Ocean University, Shanghai 201306, China; 2 Key Laboratory of Exploration and Utilization of Aquatic Genetic Resources, Ministry of Education, Shanghai Ocean University, Shanghai 201306, China; 3 School of Ocean, Yantai University, Yantai 264005, China

**Keywords:** *Eriocheir sinensis*, glycerophospholipid metabolism, transcriptome, metabolome, detoxification

## Abstract

As an important economic aquatic product in China, the farming method of *Eriocheir sinensis* significantly impacts its quality and physiological metabolism. In this study, the effects of lake (LK) farm and pond (PD) farm on the gene expression profiles and metabolic pathways in *E. sinensis* were evaluated by integrating transcriptomic and metabolomic analyses. A total of 812 differentially expressed genes (DEGs) were identified in the hepatopancreas of crabs. The DEGs were mainly enriched in nutrient reservoir activity, regulation of response to oxidative stress, and lipid transporter activity. In addition, LC-MS analysis identified 410 significantly differential metabolites, and KEGG pathway enrichment showed that these metabolites were mainly enriched in the MAPK signaling pathway, HIF-1 signaling pathway, and glycerolipid metabolism. Integrated transcriptomic and metabolomic analyses revealed that the AMPK signaling pathway, cytochrome P450-mediated xenobiotic metabolism, glycerophospholipid metabolism, and the apoptosis signaling pathway collectively exert a significant influence on the growth performance of crabs. Collectively, our findings demonstrated that the crabs in the LK group exhibit enhanced antioxidant and detoxification capacities, concomitant with reduced protein synthesis and energy metabolism, and underwent increased apoptotic events. The finding of this study will provide valuable and novel insight into crab farming practices in different aquaculture environments, providing theoretical foundations for optimizing ecological aquaculture models in Datong Lakes’ crab farms. Specifically, combined supplementation with natural feed organisms and mechanical aeration may effectively mitigate benthic hypoxia and nutritional deficits, thereby promoting sustainable production in the lake-based culture of crabs.

## Simple Summary

Distinct rearing environments and farming models exert a significant impact on the physiological metabolism and overall quality of cultured aquatic organisms. In this study, we aimed to investigate the physiological and metabolic differences in the Chinese mitten crab, *Eriocheir sinensis,* farmed in lake and pond environments using integrated transcriptomic and metabolomic analyses. The combined analysis revealed that key pathways, including AMPK signaling, cytochrome P450-mediated xenobiotic metabolism, glycerophospholipid metabolism, and apoptosis, collectively influence crab growth performance. Specifically, crabs from the lake group exhibited enhanced antioxidant and detoxification capacities. However, this was accompanied by reduced protein synthesis, lower energy metabolism, and increased apoptosis. These findings offer valuable insights for optimizing crab farming practices in different aquaculture systems.

## 1. Introduction

The Chinese mitten crab, *Eriocheir sinensis*, is a commercially important freshwater crab with a high market value in East Asian countries. As an important economic aquatic species, the *E. sinensis* industry has become the pillar of the aquaculture industry [1]. The farming of *E. sinensis* has undergone rapid development in China. It is primarily conducted through two main methods: pond-based artificial farming and lake-based natural farming. Pond-based artificial farming generally offers higher yields per unit area and relatively stable economic returns. Lake-based natural farming, due to its reliance on natural conditions, has lower yields per unit area [2]. In addition to their extensive area with comparatively good water quality, crabs in the lakes are superior to those from other waters such as fish ponds.

Aquatic organisms are highly susceptible to external environmental factors such as food sources and water chemistry [3]. The aquatic environment significantly influences key traits of farmed aquatic organisms, including their physical appearance and nutritional composition. Wild and farmed aquatic species differ notably in nutrient composition, mainly due to variations in their environmental and dietary conditions. Wild crabs in lakes typically forage for food within their natural habitat, consuming aquatic vegetation, algae, and benthic organisms [4]. Conversely, pond-raised crabs are given formulated feed to guarantee the steady growth and consistent quality of aquaculture products. This distinction in feeding practices contributes to the divergent nutritional profiles of wild and farmed crabs, which in turn affects their metabolism, flavor, and nutritional value [5].

The hepatopancreas is the largest digestive and nutrient storage organ in crustaceans, and it plays an important role in carbohydrate, lipid metabolism, and energy storage [6,7]. Metabolomics, as a well-accepted approach of high-throughput analysis, can identify the comprehensive characterization of small-molecule metabolites and provide an overview of the metabolic status and global biochemical events in a biological system. As an analytical approach, metabolomic profiling quantifies low-molecular-weight metabolites in cells or tissues, thereby offering valuable insights into the mechanisms by which organisms interact with their environment [8].

In this study, we conducted untargeted liquid chromatography–mass spectrometry (LC–MS) and RNA sequencing to investigate differences in the metabolomic and transcriptomic profiles between the crabs in the PD and LK group. The finding of this study will provide valuable and novel insight into crab farming practices in different aquaculture environments, providing theoretical foundations for optimizing ecological aquaculture models in Datong Lakes’ crab farms.

## 2. Materials and Methods

### 2.1. Animals

Healthy female crabs (average weight 171.25 ± 11.09 g) were collected from Lake Datong and adjacent ponds. Datong Lake is located at 29°05′~29°16′ N and 112°26′~112°35′ E in the nearly middle part of Hunan Province, China. It is the largest inland lake in Hunan Province. Juvenile crabs from the same family line (Jianghai 21) were reared separately in lakes and ponds. The crabs in the pond (PD) were fed a high-protein diet (91% of dry matter, 56% of crude protein, 9% of crude lipid, and 13% of ash twice per day. However, the crabs from Datong Lake (LK) were mostly dependent on natural food. After approximately nine months of rearing, eight crabs were randomly selected from each of the LK and PD groups. The hepatopancreas of each sample was immediately dissected and then stored in liquid nitrogen. Each group was divided into two parts, with one part selected for transcriptomic analyses and the other analyzed via the metabolomics platform.

### 2.2. RNA Isolation and cDNA Library Preparation

Total RNA was extracted by using TRIzol^®^ Reagent (Invitrogen, Carlsbad, CA, USA) according to the manufacturer’s instructions and the genomic DNA was removed by using DNase I (Takara, Shiga, Japan). The RNA integrity was checked by an Agilent 2100 Bioanalyzer (Agilent, Santa Clara, CA USA), while the RNA concentration was determined by an ND-2000 ultraviolet spectrophotometer (NanoDrop Technologies, Wilmington, DE, USA). The RNA-seq transcriptome library was prepared following Illumina^®^ Stranded mRNA Prep, Ligation (San Diego, CA, USA), using 1 μg of total RNA. Briefly, mRNA was enriched using Oligo d(T) magnetic beads, followed by RNA fragmentation, reverse transcription, end repair, adenylation, adapter ligation, and PCR amplification. The library was sequenced using an Illumina HiSeq 6000 (Illumina Inc., San Diego, CA, USA) with paired-end 150 bp reads. All the raw sequence data of RNA-seq have been deposited in the National Center for Biotechnology Information Sequence Read Archive (SRA) under Bio-Project accession number PRJNA1050822.

### 2.3. RNA Sequencing and Data Analysis

The raw paired-end reads were trimmed and quality controlled by fastp with default parameters [9]. was performed based on the sequence of the *E. sinensis* genome (Genbank assembly accession GCF_024679095.1) as a reference. An index of the reference genome was built and the clean reads were aligned to the reference genome using Hisat2 v2.0.5 [10]. The number of reads mapped to each gene was counted via the feature counts. Read counts were further analyzed and the edgeR was used to identify the differentially expressed genes [11]. The resulting *p*-values were adjusted using Benjamini and Hochberg’s approach for controlling the false discovery rate. The Cluster Profiler 4.6.2 R package was used to test the statistical enrichment of differentially expressed genes in the KEGG pathways [12].

### 2.4. Quantitative Real-Time PCR Validation

The accuracy of the high-throughput data was validated using qPCR methods. Ten DEGs were randomly selected from the transcriptome data, and the qPCR experiment was performed on an ABI 7500 real-time PCR system (ABI, Waltham, MA, USA). Beta-actin was used as the internal reference, and the amplifications were performed according to the following program: 95 °C for 30 s and 40 cycles of 95 °C for 5 s, 60 °C for 35 s, and 72 °C for 52 s. All primer sequences used in this study were listed in Supplementary Table S1. Gene expression levels were calculated using the 2^−ΔΔCt^ method. Statistical significance (*p* < 0.05) was calculated using one-way ANOVA and Duncan’s multiple range tests (SPSS 21.0). The minimum significant level was set to 0.05.

### 2.5. The Metabolomics Analysis of Hepatopancreas Tissues

Chromatographic separation was performed on an Acquity UPLC HSS T3 column (2.1 mm × 100 mm, 1.8 μm, Waters Corp., Milford, CT, USA). A 2 μL aliquot of the sample was separated by an HSS T3 column and then entered into mass spectrometry detection. The mobile phases consisted of 0.1% formic acid in water:acetonitrile (95:5, *v*/*v*) and 0.1% formic acid in acetonitrile:isopropanol:water (47.5:47.5:5, *v*/*v*). The mass spectrometric data were collected using a Thermo UHPLC-Q Exactive Mass Spectrometer (Thermo Scientific, Milford, CT, USA) equipped with an electrospray ionization source operating in either positive or negative ion mode. One quality control (QC) sample was run to ensure the detection stability and replicability of the samples.

### 2.6. Integrative Analysis of Metabolomics and Transcriptomics

An integrative metabolomics and transcriptomics approach was adopted to better characterize the regulation of gene expression and metabolomics. All of the differentially expressed genes and metabolites were mapped to the KEGG pathway database, their common pathway information was obtained, and a KEGG enrichment result was generated for the pathways that were significantly enriched with DEGs and DEMs.

### 2.7. Statistical Analysis

Statistical analyses were performed using the SPSS 21.0 software package. All data are expressed as means ± standard error of the means (SEM), and homogeneity of data variance was analyzed using Levene’s test. In terms of MS data analysis, unsupervised PCA was performed by statistics function prcomp within R. The data were unit variance scaled before unsupervised PCA. Differential metabolites were selected on the basis of VIP > 1, absolute Log_2_FC.

## 3. Results

### 3.1. Transcriptomic Analysis of E. sinensis in the LK and PD Group

**Figure 1:**
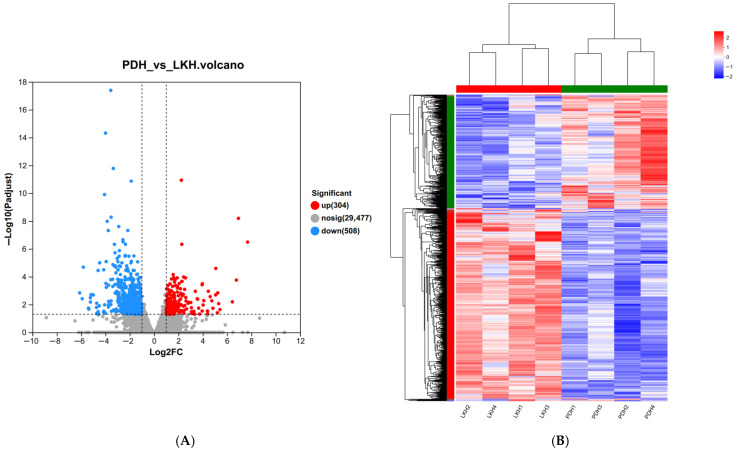
The volcano plot (**A**) and heatmap (**B**) of differential expressional genes (DEGs) in the LK and PD crabs. Red color indicates up-regulated genes and blue color means down-regulated genes between groups.

The criterion of *p*-value < 0.05 and |log_2_ FC| > 1 was used to identify differentially expressed genes (DEGs) in the LK group and the PD group. A total of 812 DEGs were identified in the LK vs. PD group, of which 304 DEGs were significantly up-regulated and 508 DEGs were significantly down-regulated in the LK group (Figure 1A, Supplementary Table S2). A hierarchical clustering analysis was conducted on differentially expressed genes, revealing distinct patterns of gene expression differentiation between the PD group and the LK groups (Figure 1B).

**Figure 2:**
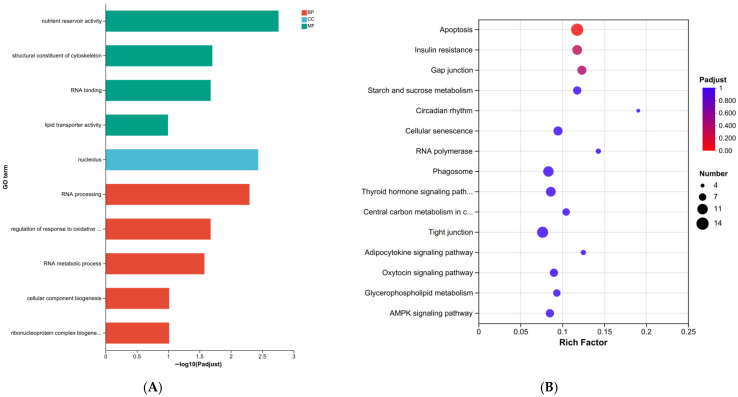
(**A**), Gene ontology (GO) enrichment of DEGs in the LK and PD crabs. Red represents biological process (BP), blue represents cellular component (CC), and green represents molecular function (MF). (**B**), KEGG pathway analysis for DEGs in the LK and PD crabs. The *X*-axis represents the enrichment factor for each of the differentially expressed genes in each pathway. The *Y*-axis shows the name of the enriched pathway. The size of each node represents the number of enriched genes, and p.adjust values are indicated by changing colors moving from red to blue.

These DEGs were subjected to GO enrichment and KEGG functional analysis to better understand the biological significance of these DEGs and the biochemical processes involved. The top 20 GO enrichment terms indicated that the DEGs were mainly involved in some important biological processes and molecular functions, such as nutrient reservoir activity, regulation of response to oxidative stress, and lipid transporter activity (Figure 2A). In addition, the results of the KEGG enrichment analysis showed that some pathways, such as the glycerophospholipid metabolism, linoleic acid metabolism, and starch and sucrose metabolism, were enriched (Figure 2B).

### 3.2. Quantitative Real-Time PCR Validation of the DEGs

**Figure 3:**
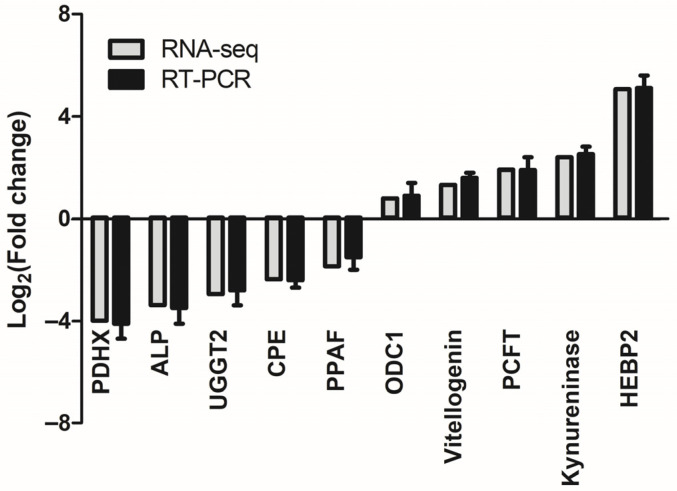
The expression of 10 DEGs in the transcriptome was verified by qRT-PCR. The *X*-axis indicates the gene names; the *Y*-axis of the columns in the chart represents the value of log_2_ (fold change).

To confirm the reliability of the transcriptome sequencing results, we selected 10 DEGs for quantitative real-time PCR (qRT-PCR) validation. They included five up-regulated genes and five down-regulated genes. The results showed that the qRT-PCR gene expression patterns were consistent with the RNA-seq results (Figure 3), which demonstrated the reliability and accuracy of RNA-seq.

### 3.3. Overview of Metabolomic Profiles from Crabs in the LK and PD Group

**Figure 4:**
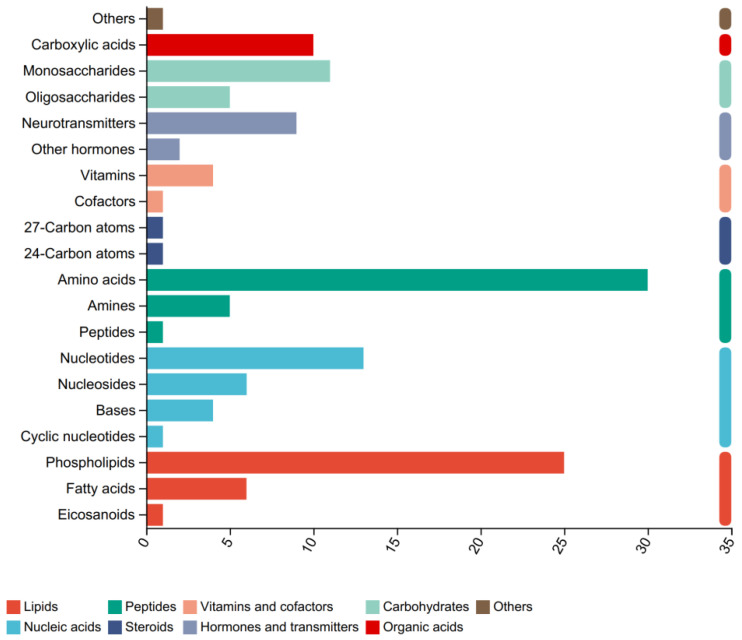
Overviews of metabolic profiles based on chemical taxonomy.

Using molecular weight and mass spectrometry information, a search was conducted of the HMDB to identify metabolites. A total of 410 metabolites, including amino acids, carboxylic acid, monosaccharides, nucleotides phospholipids, and fatty acids, were identified in the hepatopancreas metabolomic profiles (Figure 4). The analysis revealed that the top three super classes among the identified metabolites were amino acids (29.18%), phospholipids (24.36%), and nucleotides (14.41%).

**Figure 5:**
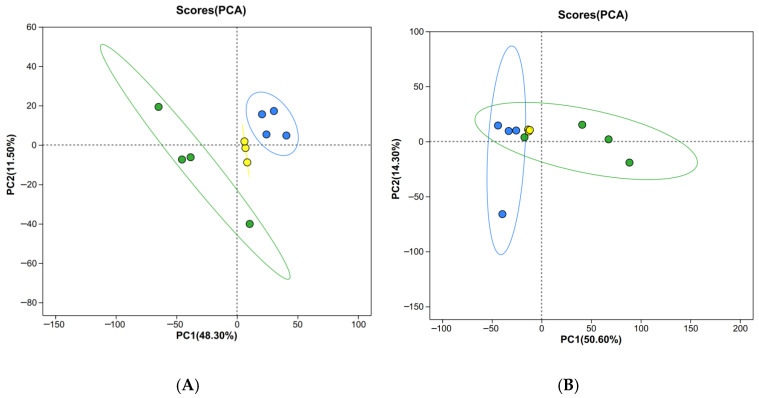
The PCA scores plot of samples acquired in the positive (**A**) and negative (**B**) ion mode. Green spots mean the PD group, blue spots mean the LK group, yellow spots mean QC.

**Figure 6:**
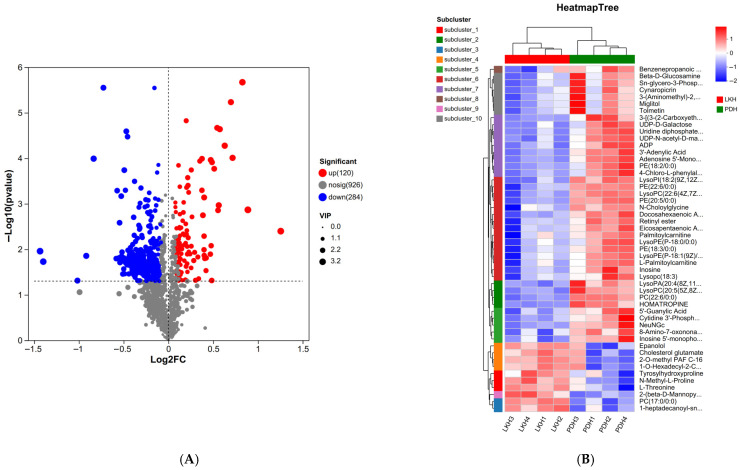
(**A**), the volcano map of DEMs in the LK and PD crabs. (**B**), hierarchical clustering analysis for DEMs and the metabolomics between the LK group and the PD group. Red and blue indicate metabolites up-regulated and down-regulated in LK group.

To better understand the metabolic differences in the two groups, PCA was applied for pairwise comparisons. In positive (Figure 5A) and negative (Figure 5B) ion modes, there was an apparent separation observed in the crabs between the LK and PD groups. The percentage of explained value in the metabolomics analysis of PC1 and PC2 was 48.30% and 11.50% (positive ion mode), and 50.60% and 14.30% (negative ion mode), respectively. On the basis of the PCA results, a total of 410 significantly differential metabolites (SDMs) were identified between the PD and LK groups. Among the 410 SDMs, 292 and 118 metabolites were significantly up-regulated and down-regulated compared with those in the PD group (Figure 6A). Hierarchical clustering analysis also indicated that each type of the two groups exhibited a distinct metabolic pattern (Figure 6B), and the detailed metabolite identification and quantification results can be found in Supplementary Table S3.

**Figure 7:**
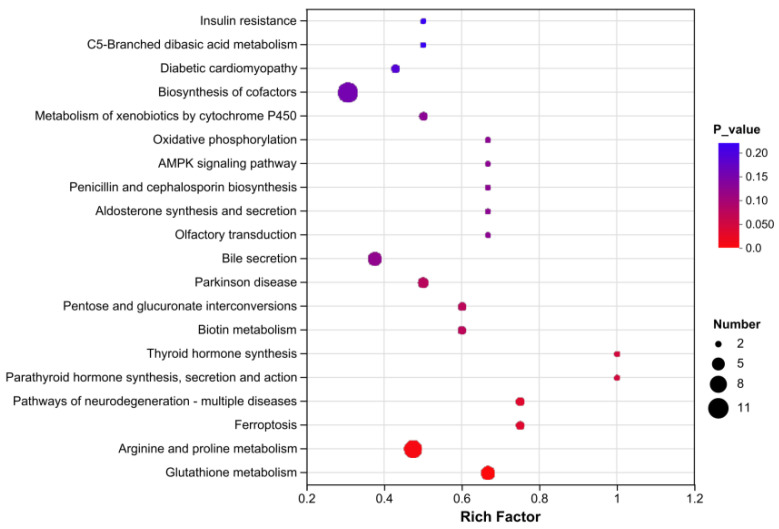
KEGG pathway analysis of DEMs in the two groups. The vertical coordinates indicate the top 20 terms, and the horizontal coordinates indicate the enrichment factor.

KEGG enrichment analysis was performed on these SDMs to explore the most relevant pathways. The results showed that the top enriched pathways were mainly enriched metabolite categories, including the Arginine and proline metabolism, AMPK signaling pathway, HIF-1 signaling pathway, and glycerolipid metabolism (Figure 7).

### 3.4. KEGG Pathway Enrichment Analysis Based on Metabolomics and Transcriptomic Data

**Figure 8:**
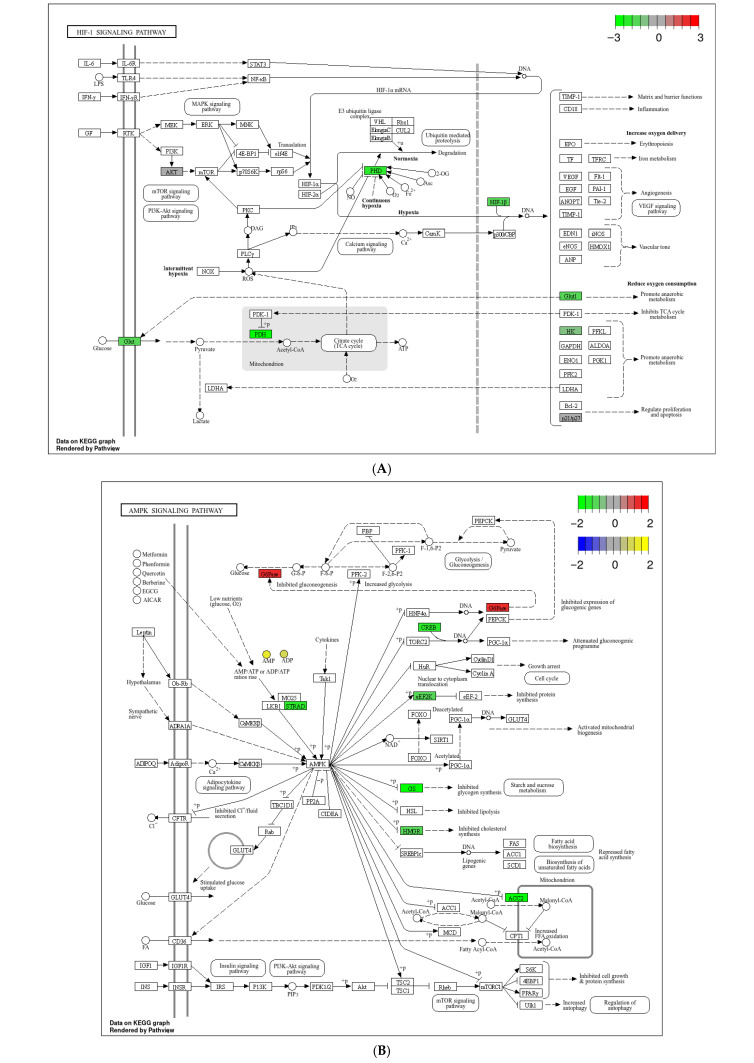

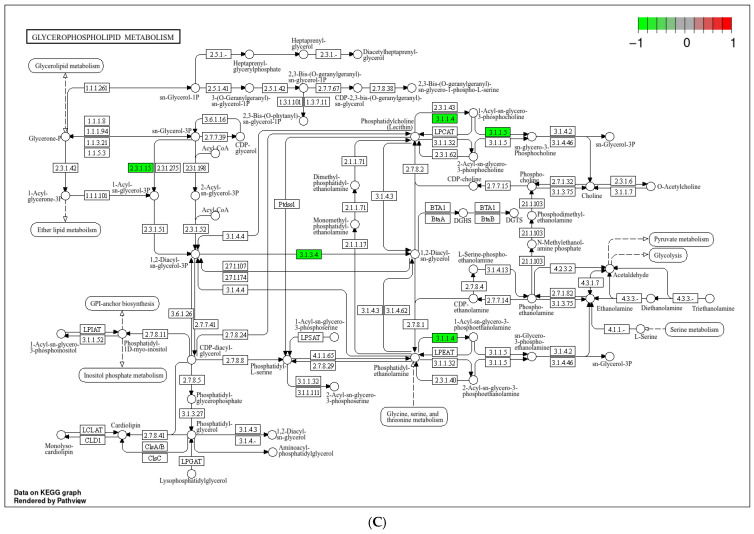
Classification of DEGs and DEMs in pathways through integrated analysis. (**A**), HIF-1 signaling pathway. (**B**), AMPK signaling pathway. (**C**), glycerophospholipid metabolism pathway.

To further screen related genes and metabolites, as well as key metabolic pathways, we conducted a comprehensive analysis of the transcriptome and metabolome. Analysis of DEGs and DMs in the two groups showed that the HIF-1 signaling pathway (Figure 8A), AMPK signaling pathway (Figure 8B), and glycerophospholipid metabolism pathway were enriched in the KEGG analyses (Figure 8C).

## 4. Discussion

Prior to 2017, extensive aquaculture practices characterized by high-density stocking and intensive feeding significantly damaged the original aquatic vegetation in Datong Lake, resulting in the near-complete loss of its self-purification capacity. In recent years, under governmental support and technical guidance from expert teams, 80% of the area of Datong Lake was successfully recovered by submerged vegetation, and the water quality was improved [13]. Datong Lake has pioneered an innovative “macrophyte + Chinese mitten crab” ecological development model. Although the natural lake aquaculture of crabs eliminates feeding costs and simplifies management protocols, it presents several significant operational and environmental challenges.

### 4.1. The Activation of Detoxification and Antioxidant Genes in the LK Group

Uridine diphosphate (UDP)–glycosyltransferases, which are major detoxification enzymes, play an important role in the glycosylation of lipophilic endobiotics, xenobiotics, and phytoalexins [14]. Glucosylation by UDP–glucosyltransferase is crucial for reducing the autotoxicity of plant allelochemicals [15]. Submerged plants and their associated periphyton serve as food sources for zoobenthos. In turn, zoobenthos are consumed by crabs, indirectly providing an essential food supply for crabs [16]. Previous studies have found the presence of both macrophytes and algae in gastric mills, indicating that crabs consume host plants and algae [17]. Saxitoxin, which is derived from the mollusc Saxidomus giganteus, is mainly produced by toxic dinoflagellates [18]. Cytochromes P450 (CYPs) are a major class of enzymes responsible for xenobiotic metabolism. In this study, the expression level of cytochrome P450 was significantly up-regulated in the LK group. The metabolome data also revealed the presence of toxic phenylpropanoids and polyketides, such as saxitoxin, further supporting this hypothesis. These findings are consistent with previous research on Praeruptorin, a bioactive compound isolated from the traditional Chinese medicinal herb Qianhu (*Peucedanum praeruptorum Dunn*). Specifically, Huang et al. (2013) demonstrated that Praeruptorin treatment significantly enhanced both the mRNA expression and enzymatic activity of cytochrome P450 enzymes in LS174T cells [19].

In addition, GO enrichment analysis found pathways related to the term of response to oxidative stress. The term included two genes, *LOC127009260* (*sestrin-2like*) and *LOC126988476* (*nucleoside diphosphate kinase 5-like*). The gene *sestrin 2*, a stress-inducible protein associated with various stress conditions, is a potential antioxidant [20], both of which are significantly up-regulated in the LK group, suggesting that the cellular capacity for detoxification and antioxidation is significantly enhanced in the LK group [21,22].

### 4.2. Enzyme Involved in Digestion in the Hepatopancreas of Crabs

For carbohydrate metabolism, the amylase activity in the hepatopancreas of crabs in the LK group was significantly lower than that in the PD group. In a natural lake environment, crabs are omnivorous and feed on a variety of food sources. However, in pond aquaculture, many sources of carbohydrates have been added to the aquafeed, such as maltose, sucrose, and wheat starch [23]; hence, the amylase in the hepatopancreas was activated to accommodate higher dietary carbohydrate [24,25]. In addition, metabolome analysis also revealed that the levels of oxoglutaric acid in the hepatopancreas were significantly up-regulated in the PD group, indicating that the energy metabolism level of crabs in the PD group was significantly higher than that in the LK groups.

Similarly, with regard to protein digestion, carboxypeptidase B was up-regulated in PD crabs, suggesting an increased capacity for protein digestion, which is also consistent with the higher protein content in the consumed diet in the PD crabs. The hepatopancreas functions as the central metabolic organ in organisms, and its enzyme activity exhibits dynamic regulation in response to dietary composition [26,27]. Considering that crab in the pond farm consumed artificial feed, which is rich in carbohydrate and protein [28], it was reasonable that the digestion levels of protein and carbohydrate levels were higher in the PD groups. Other researchers have also reported similar results. For instance, digestive enzymes have shown a positive correlation between protease, amylase activities, and dietary protein levels [29,30].

Vitellogenin (Vtg) is a key supplier of nutrients and energy during the development procession in crabs [31]. In this study, the expression of *vitellogenin* mRNA was significantly lower in the LK group than that in the PD group [32]. Similarly, there were lower levels of VgA and VgB mRNAs in wild fish compared to captive individuals [33].

### 4.3. Regulation of Energy Metabolism and Apoptosis Procession

The TCA cycle is the final metabolic pathway of the three major nutrients and is also the hub of the carbohydrate, lipid, and amino acid metabolisms [34]. In this study, we found that two metabolites (ADP, AMP) and key genes (*cyclic AMP-dependent transcription factor ATF-6 alpha, GTP-binding protein 4*) involved in the AMPK pathway were dysregulated in the LK group. This indicates that the crabs in the LK group receive a lower energy supplement compared to those in the PD group, which is also consistent with the fact that crabs in the LK group mainly fed on mussel and phytoplankton in natural environments [35].

Apoptosis is a process of programmed cell death and is responsible for tissue remodeling and normal organization. In this study, several genes related to apoptosis procession were found, including *caspase1*, *caspsase droc*, *DDIT4,* and *NFIL3* genes. The genes *caspase1* and *caspsase dronc* were activated by inflammasomes [36] and stress-induced apoptosis [37,38]. *DDIT4* is expressed under stress situations triggered by the mTOR [39], hypoxia [40], and energy depletion [41]. Similarly, *NFIL3* has been identified as a crucial regulator involved in various cellular processes, including the immune response and apoptosis [42]. In this study, the expression of genes associated with apoptosis, such as *caspase-1*, *caspase-8* (*dronc*), and *DDIT4*, was significantly up-regulated, indicating that apoptotic events may have occurred in the LK group.

### 4.4. The Role of Glycerophospholipid Metabolic Pathway in the Response of Crabs in LK Group Against Hypoxic Stress

Multiomics analyses in multiple species including yeast, zebrafish, and mice revealed the conserved involvement of the glycerophospholipid metabolism in hypoxic adaptation [43]. For example, up-regulation of lysophospholipid acyltransferase 1 (LPCAT1), a key enzyme in dipalmitoyl phosphatidylcholine (DPPC) biosynthesis, was observed in yeast under hypoxic conditions [44]. This metabolic adaptation increased membrane DPPC levels, which demonstrated protective efficacy, as evidenced by comparable hypoxia-resistance phenotypes in mammalian cell models. Rats were subjected to chronic hypoxia for 40 days, and metabolic pathway analysis revealed that the glycerolipid and glycerophospholipid metabolism were the most significantly impacted pathways [45]. In this study, the HIF-1 signaling pathway was found in the crabs of the LK group. In static aquaculture lakes, the dissolved oxygen in the hypolimnion cannot be adequately replenished due to thermal stratification [46]. When the aquaculture density in the lake is too high, hypoxia events may occur. In agreement with these findings, the glycerophospholipid metabolism was observed in the KEGG enrichment analysis in this study, which suggested a protecting role of the glycerophospholipid metabolism in the LK crabs under hypoxic conditions.

## 5. Conclusions

Integrating transcriptomics and metabolomics provides novel insights into the molecular mechanisms underlying the significant differences in transcripts and metabolites of crabs in the LK and PD groups. The crabs in the LK group exhibit a higher capacity for antioxidant and detoxification functions, while demonstrating a lower capacity for protein synthesis and energy metabolism, and undergoing apoptotic events. Our results indicate that the different types of culture environments (LK and PD) affect the metabolism of the hepatopancreas of *E. sinensis*. This study contributes to the general understanding of the physiology of crabs in natural lakes and provides a theoretical basis for the better development of crab aquaculture in lake farming. Specifically, combined supplementation with natural feed organisms and mechanical aeration may effectively mitigate benthic hypoxia and nutritional deficits, thereby promoting sustainable crab production in lake systems.

## Data Availability

The transcriptomic data generated in this study have been deposited in the NCBI Sequence Read Archive (SRA) under BioProject accession number PRJNA1050822.

## Supplementary Materials

The following supporting information can be downloaded at: https://www.mdpi.com/article/10.3390/biology15020110/s1, Table S1. Primer sequences for the genes selected for qRT-PCR. Table S2. The differentially expressed genes (DEGs) in the PD and LK group. Table S3. The differentially expressed metabolites (DEMs) in the PD and LK group.
