# Supplementary material for: Integrative Application of Transcriptomics and Metabolomics Reveals Molecular Insight into Metabolomic Variations in Chinese Mitten Crab Eriocheir sinensis Harvested from Lake Datong and Adjacent Pond
Source: Biology (Basel). 2026 Jan 6;15(2):110. doi: 10.3390/biology15020110 (PMC12838152; doi:10.3390/biology15020110)
Supplement: Supplementary file 1 [file biology-15-00110-s001.zip › Supplement Table S1.pdf]

Supplementary Table 1. Primer sequences for the genes selected for qRT-PCR.

Gene name	Forward	Reverse
PDHX	F:GGCGAGGACTGGAAGACTGT	R: CTGCCCTGAGGCCATACTG
ALP	F:GGCGAAGAGCGACTGTAA	R: TTCCTCTGCTCCAAGTTGTC
UGGT2	F: TGGTCAACTCCCACTATTCCC	R: AACGCTTCCACCAACGACT
CPE	F: CAAGGGCGGCGTCACTAA	R: ATGATGGCGTTGGGCAGA
PPAF	F: GGAGGAATGCTGGTGGAGTG	R: CGAGTCTGAGTAGGCGGGTTT
ODC1	F: CAATGATGGCGTCTATGGG	R: GATGGAGCAGGGCAAGTAA
Vitellogenin	F: ATCTACTCAGCCGCATTCTAC	R: CAGGTCGTTCTGGATCATTT
PCFT	F: GCCGCGTCAACCTCAACTT	R: GCGTCTCGGGCGTAGTGAT
Kynureninase	F: CAGAGCCATGCGAGACAGATT	R: AGTCGTCCAACACCTCCAAGA
HEBP2	F:GGGTTACCACATCCTTCCTCT	R: TTTCACAGTTGGGTCCTTCG
